# Phantom Sensations Influenced by Global and Local Modifications of the Prosthetic Socket as a Potential Solution for Natural Somatosensory Feedback During Walking: A Preliminary Study of a Single Case

**DOI:** 10.3389/fresc.2022.803912

**Published:** 2022-02-23

**Authors:** Lisa Bachini, Stéphane Liszez, Serge Mesure, Claire Mahé, Amélie Touillet, Isabelle Loiret, Jean Paysant, Jozina B. De Graaf

**Affiliations:** ^1^Aix Marseille Univ, CNRS, ISM, Marseille, France; ^2^Lagarrigue Orthopédie, Centre Prothétique Houradou, Marseille, France; ^3^IRR Louis Pierquin, UGECAM Nord-Est, Nancy, France

**Keywords:** amputation, lower limb, referred phantom sensations, prosthetic socket, sensory feedback, gait, interface

## Abstract

Following lower limb amputation, amputees are trained to walk with a prosthesis. The loss of a lower limb deprives them of essential somatosensory information, which is one of the causes of the difficulties of walking with a prosthesis. We here explored whether a solution to this lack of somatosensory feedback could come from natural sensations of the phantom limb, present in most amputees, instead of from substitutive technologies. Indeed, it is known that phantom sensations can be modulated by (i) global mechanical characteristics of the prosthesis socket, and (ii) locally applying a stimulus on an area of the residual limb. The purpose of this pilot study was to verify the feasibility of influencing phantom sensations *via* such socket modifications in a participant with transfemoral amputation. Four prosthetic interface conditions were studied: a rigid and a semi-rigid socket, each one with and without a focal pressure increase on a specific area of the residual limb. The results show that phantom sensations during walking were different according to the 4 interface conditions. The participant had more vivid phantom sensations in his foot and calf of which some varied as a function of the gait phases. Preliminary gait analysis with wearable sensors shows that these modifications were accompanied by changes in some gait spatiotemporal parameters. This preliminary study of single case demonstrates that phantom sensations can be modulated by the prosthetic interface and can provide natural somatosensory information dynamically varying with gait phases. Although this needs to be confirmed for a larger population of lower limb amputees, it already encourages non-painful phantom sensations to be considered early during the rehabilitation of lower limb amputees.

## Introduction

### Background

Walking is heavily based on somatosensory feedback that informs about the state of the body and its interactions with the environment (1). Indeed, skin afferents from the plantar sole (2–5) and muscle and joint proprioceptive information (6, 7) are crucial for maintaining balance. After lower limb amputation, one is forced to walk with a prosthesis, thus without most of this feedback. Walking, an almost automatic behavior in a non-amputee, becomes a tiring activity that requires significant cognitive resources (8–10). Moreover, this lack of somatosensory information could be one of the causes of the asymmetry (8, 11) characterizing the gait of amputees (12–14). Asymmetry is probably involved in the increased prevalence of degenerative pathologies of the contralateral limb (15–17). Therefore, the loss of lower limb somatosensory feedback impacts daily activities and could cause long-term health problems.

The provision of somatosensory information on the position of the prosthesis or ground characteristics has been found to improve the efficiency of prosthetic walking. Through implanted electrodes, a team of researchers stimulated the peripheral nerves of the residual limb to give feedback about pressure detected by sensors on the prosthetic plantar sole and about the flexion angle of the prosthetic knee (18). Many variables related to prosthetic gait efficiency (cognitive load, oxygen consumption, walking speed, number of falls) were improved by this feedback, which is promising. Other work has demonstrated the value of providing somatosensory information using a non-invasive technique by instrumentation of the prosthesis alone. For example, after a training period, cutaneous vibratory stimulation—when consistent with the phases of the prosthetic stance phase—reduced temporal gait asymmetry and increased stride length without increasing cognitive load (11). These studies suggest that additional information about gait phases leads to more efficient prosthetic walking. However, these studies used invasive or instrumented solutions that are non-intuitive and therefore need a training period. The present study consisted in exploring a new solution allowing intuitive somatosensory feedback during walking through naturally present phantom limb sensations.

A majority of amputees still perceive the lost limb through a natural phenomenon called “phantom limb” (19–21). These phantom sensations are often non-painful, manifesting as tingling, warmth, or a simple sense of presence as an intact limb (19, 22). Painful phantom sensations also exist but, contrary to popular belief, are not as common as non-painful sensations and are often occasional and moderate (21). Non-painful phantom sensations during dynamic and functional activities such as walking have only been little reported in the literature. Yet, according to our recent interviews with 97 lower limb amputees, 30% of the participants report perceiving their phantom limb when walking. Moreover, the phantom limb can not only be present during walking, but even interact with it because it were perceived as either indispensable, as an aid, or sometimes as a disturbance. This suggests that these natural sensations may compensate for the lack of somatosensory information if they are coherently varying with the gait phases. Interestingly, in some amputated persons, stimulation of the residual limb modulates phantom sensations. These sensations are called referred sensations (RS) (22, 23). One can distinguish local and global stimulation. Local stimulation [static or dynamic pressure (23, 24), vibration (25), or electrical stimulation (26)] applied directly on specific areas of the skin can evoke changes in phantom sensations in some amputees. Regarding global stimulation, we found that for many participants, wearing their prosthetic limb, even without seeing it, changed their phantom sensations. Thus, global pressure applied on the residual limb by contact with the prosthetic socket seems to be a form of stimulation that influences phantom sensations. This is interesting because local stimuli in the socket or global stimuli related to the socket itself could be exploited to induce RS that vary coherently with the gait phases and would thus be usable as a form of somatosensory feedback.

### Objectives

The main objective of this preliminary study was therefore to investigate the feasibility of modifying the phantom sensations perceived during walking by intervening on the prosthetic interface of a lower limb amputee participant. To study whether the different interface conditions also influenced the gait pattern of the participant, a preliminary analysis of some gait parameters was performed.

## Methods

### Study Design

This study is a single case cross-sectional study since we observed and analyzed the effect of an intervention on the prosthetic socket at a specific point in time.

### Participant

The participant was 48 years old and had a traumatic transfemoral amputation of his left leg 5 years before the present study. He daily wore a prosthesis with an adherent semi-rigid socket without a sleeve, could walk long distances, and did not take medication. He reported neither phantom nor residual limb pain, and he was able to describe his phantom sensations in detail. Written informed consent was obtained before the study.

### Setting

This study was conducted in 4 phases, all in the Chantecler Rehabilitation Center (Marseille, France) and in the presence of medical staff. At the end of March 2021, a semi-structured interview regarding the participant's phantom sensations was conducted. Then, the participant was seen 3 times during April 2021, first to perform the mapping of RS, then to design the 4 prosthetic interface conditions, and finally, to make the participant walk with the 4 interfaces to analyze his phantom sensations during walking and some gait spatiotemporal parameters.

#### Phase 1: Exploration of Phantom Limb Sensations With a Semi-structured Interview

The semi-structured interview allowed for a detailed description of non-painful and painful phantom sensations as well as factors influencing them. Phantom sensations were described according to their nature (e.g., tingling, touch, heat, “muscular contraction”) and their location (e.g., toes, heel, calf). Furthermore, we asked the patient to describe the factors influencing them (e.g., weather, fatigue, activity), and explored, in particular, the influence of different conditions: sitting with and without a prosthesis, standing with a prosthesis, and while walking. The interview was completed by a clinical examination to search for RS by palpation of the residual limb. The interview lasted 1.5 h.

#### Phase 2: Mapping of Referred Sensations

**Figure 1:**
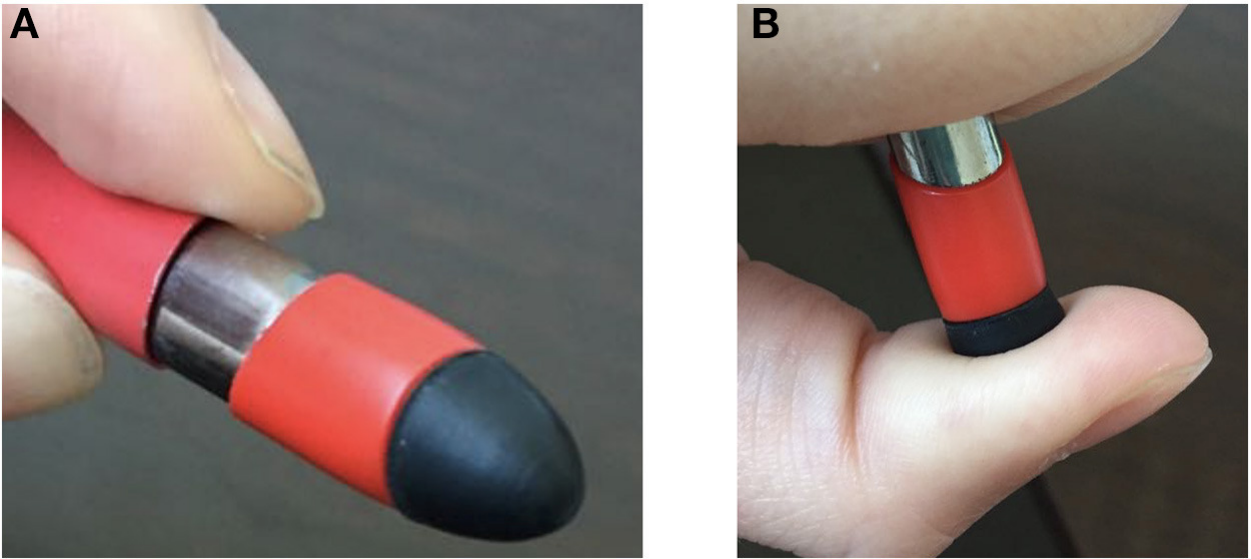
The instrument used to explore referred sensations (RS) by stimulating different areas with the soft rubber tip **(A)**, pressing on the skin **(B)**. The participant reported RS during intense pressure.

**Figure 2:**
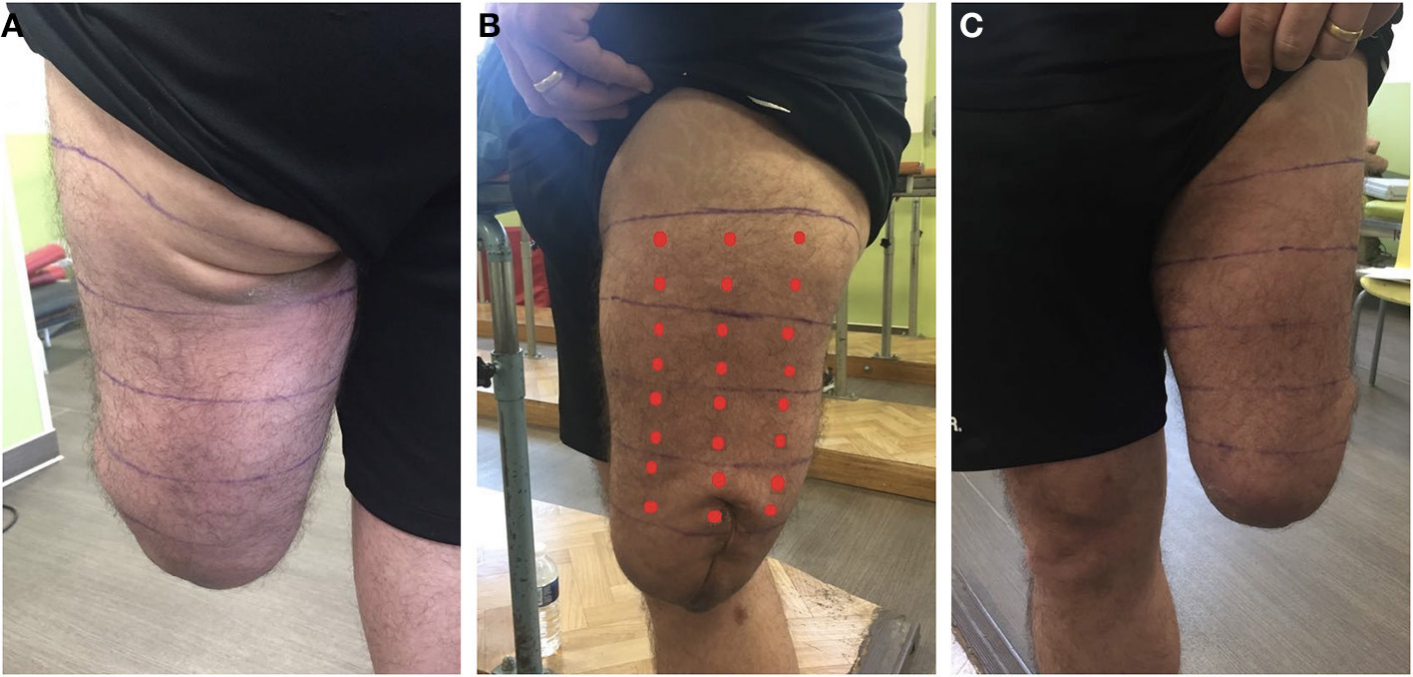
Posterior **(A)**, lateral **(B)**, and anterior **(C)** views of the residual limb showing the 4 horizontal strips drawn for mapping. The most proximal horizontal delimitation was placed just under the groin and drawn perpendicular to the vertical axis of the residual limb. In each strip, 24 points around the leg were tested. The red dots in B represent the distribution of the stimulated points on one view. The part of the residual limb below the most distal strip was not tested as it was numb.

As the interview showed that the participant had RS, a detailed mapping was carried out to identify both the areas of the residual limb for which stimulation could modify phantom sensations and the nature of the RS. Intense localized pressure on the skin appeared to induce RS in the phantom limb (Figure 1). A total of 69 areas of the residual limb were defined by dividing the residual limb into 4 faces (medial, lateral, posterior, and anterior), each divided into 4 horizontal strips of 5 cm high, each comprising 6 areas to be stimulated (Figure 2). The participant was standing during the mapping.

#### Phase 3: Global and Local Modifications of the Prosthetic Interface

**Figure 3:**
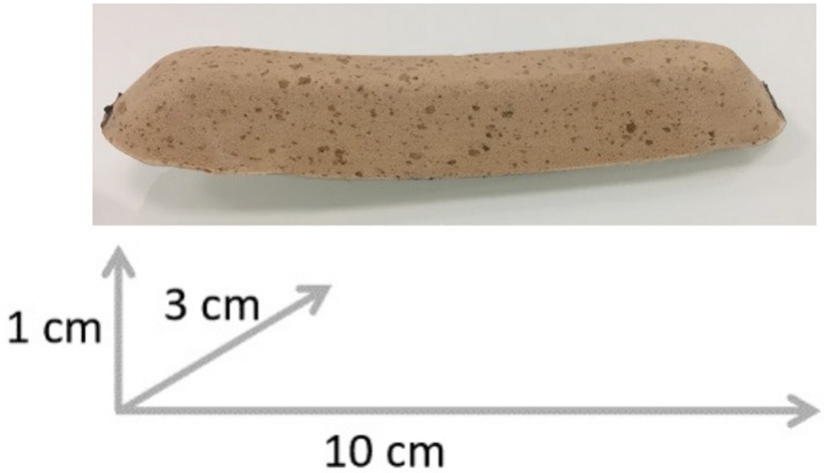
The cork patch and its dimensions used to locally modify the prosthetic interface. It was 10 cm long, 3 cm wide, and 1 cm thick, rounded on the edges that touched the skin to avoid high pressure on the skin at these edges.

Based on the RS map, a patch was designed and placed inside the socket to apply pressure on skin areas that induce RS potentially useful for walking, thereby locally modifying the prosthetic interface (Figure 3). Furthermore, to study the influence of global changes of the prosthetic interface, two types of sockets made of different materials were used: rigid plastic called glycolic polyester (PETG) and semi-rigid plastic called ThermoLyn® that is surrounded by carbon support. The form and dimensions of these two sockets were the same, as were the alignments of all prosthetic components. The rigid socket had already been worn daily by the participant as it had been his temporary socket.

#### Phase 4: Description of the Phantom Limb Sensations When Walking and Recording of Gait Parameters for Each of the 4 Interface Conditions

To study whether the modifications of the prosthetic interface modified the phantom sensations, the participant was asked to walk for 2 min on a treadmill for each interface condition and then to describe in detail his phantom sensations. A treadmill protocol was preferred to an over-ground walking protocol to control the speed and optimize the regularity of the walking cycles. Preferred speed was determined on the ground during a timed walk over a distance of 10 m while wearing the semi-rigid socket without the patch. For safety reasons, the treadmill walking speed was set at 80% of the preferred speed, and the participant had to hold on lightly to the bars of the treadmill. The same speed (i.e., 3 km/h) was used for all conditions. The conditions were, in order: rigid socket without a patch, rigid socket with a patch, semi-rigid socket without a patch, and semi-rigid socket with a patch. A break of 10 min was taken between conditions. To study whether spatiotemporal parameters were modified by the different conditions, two Physilog® (Gait Up®) inertial units were placed on the lateral sides of the participant's shoes. The recording frequency was 128 Hz.

### Variables

**Figure 4:**
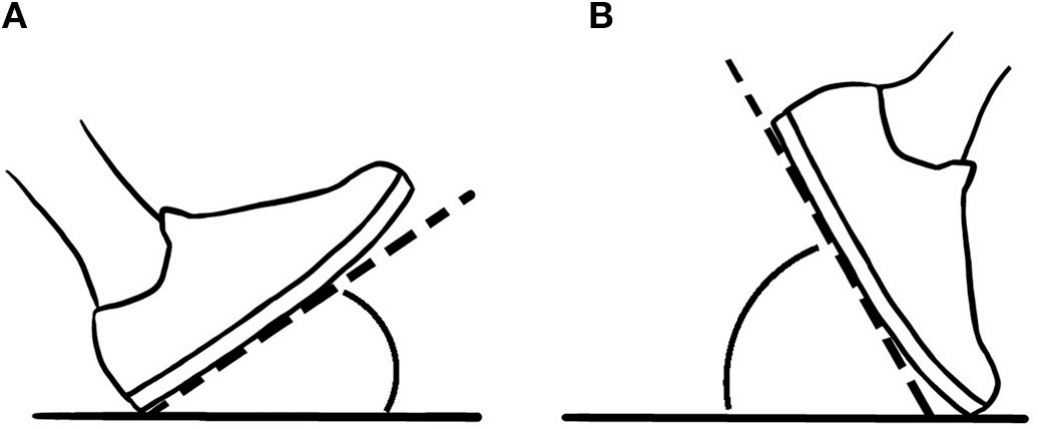
Illustration of **(A)** heel strike angle and **(B)** toe-off angle. The heel strike angle is the angle between the ground and the foot when the heel landed on the ground at the beginning of the stance phase. The toe-off angle is the angle between the foot and the ground when the toes come off at the end of the stance phase.

The phantom sensations were described qualitatively by the participant according to their nature and their location. Different gait parameters were analyzed for both the prosthetic and the contralateral limb: heel strike (Figure 4A) and toe-off (Figure 4B) angles, and duration of the double support phase. The latter corresponded to the percentage of the total duration of a gait cycle that both feet were on the ground. The first 3 gait cycles as well as the last minute of recording were removed from the data analysis, so a total of 38 gait cycles were analyzed for each interface condition.

### Study Size

In this study, the main objective was to show that it is possible to modify the phantom sensations of an amputee by intervening on the socket, either locally or globally. This is an exploratory study to investigate the feasibility of this method of modifying phantom sensations with a single participant, before developing a larger scale study based on this method. Therefore, only one participant was included.

### Statistical Methods

R studio software (Version 1.3.1) was used to perform statistical testing on the gait parameters. As the data were from the same participant, a permutation ANOVA (27) was performed with 2 factors (type of socket and presence of patch).

## Results

### Descriptive Data

#### Phase 1: Exploration of Phantom Limb Sensations With a Semi-structured Interview

**Figure 5:**
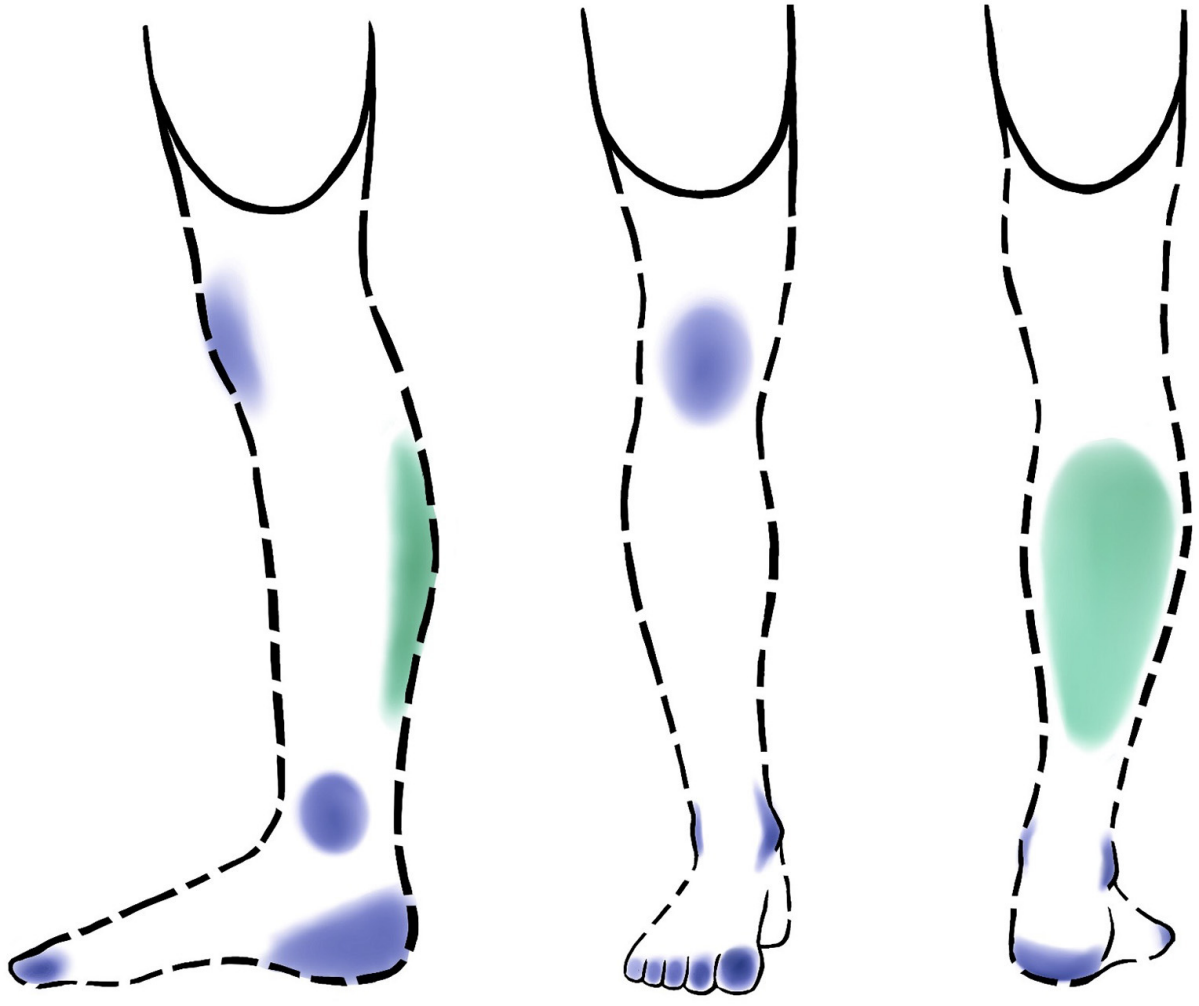
Location of the participant's phantom sensations and the nature of the sensations (green: muscle contractions; blue: tingling) in a sitting position and without a prosthesis. In the standing position with a prosthesis and when walking, the areas are perceived more intensely and the locations are similar, except for the knee which is no longer perceived. Solid outline: residual limb; dashed outline: amputated limb.

The participant had permanent non-painful phantom sensations since his amputation. In a **seated position** without his prosthesis, he described permanent phantom sensations in the foot with tingling in the toes (especially the hallux), the heel, and the ankle (Figure 5). The knee was perceived occasionally and not very intensely. The participant perceived global “muscular contractions” in his calf. He did not perceive the anterior part of the leg. When **seated wearing his usual prosthesis** with the semi-rigid socket, besides the knee that disappeared, these phantom sensations remained permanent but intensified, especially for the toes. When **standing and walking**, this intensity again increased. Overall, these results show that the phantom sensations were influenced by wearing the prosthesis and the pressure applied on the residual limb.

At the end of the interview, the eventual presence of RS was explored by palpation of the residual limb. When an area on the posterior side of the residual limb was pressed, the participant felt his phantom calf more intensely. Another area was found on the medial side on which pressure induced perception of his phantom foot arch, whereas he had not perceived it spontaneously. A more detailed exploration was therefore carried out in Phase II to map his RS more methodically.

#### Phase 2: Mapping of Referred Sensations

**Figure 6:**
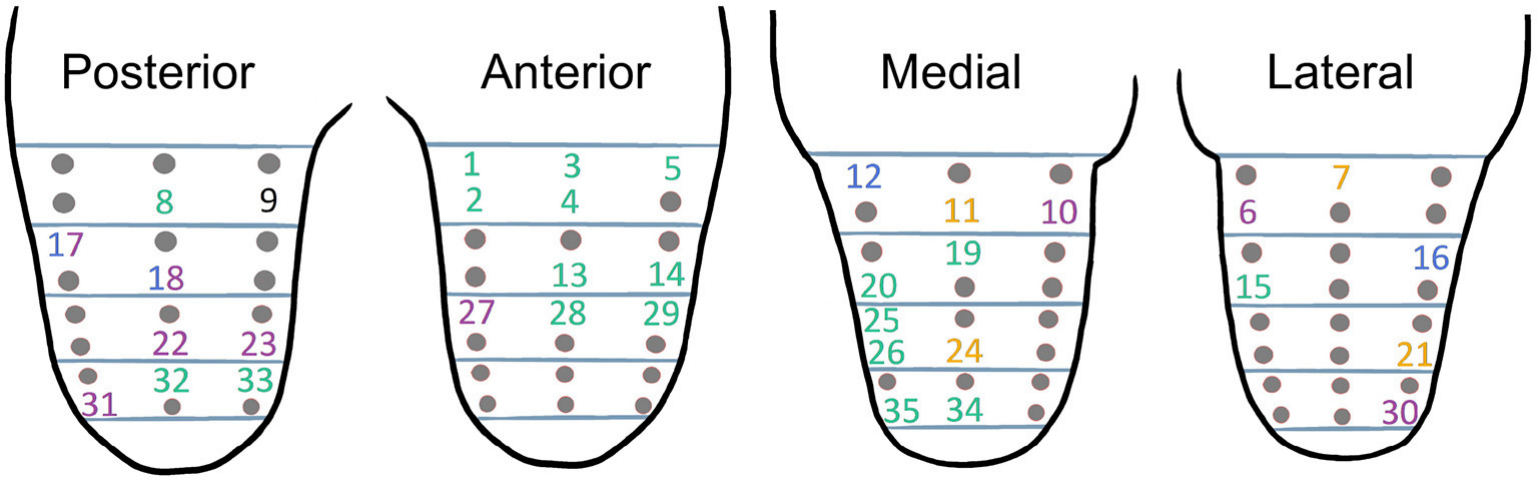
Mapping of referred sensations (RS) on 4 sides of the residual limb (from left to right: posterior, anterior, medial and lateral side). The grey dots indicate the areas that did not give rise to the RS; the numbers those that did. The color of the numbers indicates the nature and the location of the RS (green: contraction of the calf; yellow: influx in the calf; blue: tingling in the foot; purple: influxes in the foot). Pressure on area 9 evoked a sensation that was too brief and weak to be identified, and on areas 17 and 18 an impulse and tingling in the foot.

Phantom sensations were modified by pressure for 35 of the 96 stimulated areas, 9 were localized posterior on the residual limb, 10 anterior, 10 medial, and 6 lateral (Figure 6). The RS were more intense and precise localized muscle contractions in the calf and more intense tingling in the hallux, sensations on phantom segments that the participant already perceived. In addition, he perceived “influxes” in the calf and the foot, as well as tingling in the arch, dorsum, and medial side of the foot, the latter being sensations and phantom segments that the participant had not perceived spontaneously.

### Main Results

#### Phase 3: Global and Local Modifications of the Prosthetic Interface

**Figure 7:**
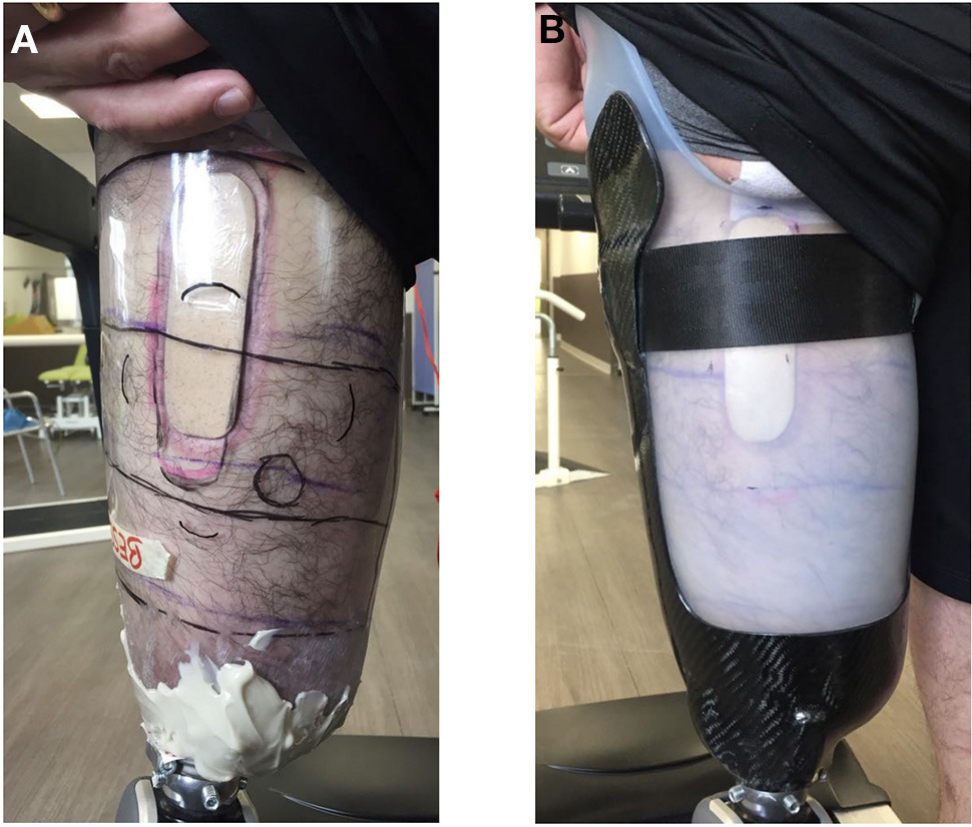
The cork patch placed on the posterior side of the rigid **(A)** and semi-rigid socket **(B)** on stimulation areas 8 and 18 (see Figure 6).

The patch was placed in both the rigid and semi-rigid sockets on areas 8 and 18 of the map (posteriorly and proximally on the residual limb, Figure 7) for which pressure induced modification of sensations of calf muscle contractions, and foot influxes and tingling in the arch of the foot (see Figure 6).

#### Phase 4: Description of the Phantom Limb Sensations When Walking and Recording of Gait Parameters for Each of the 4 Interface Conditions

Concerning the phantom sensations, for the foot, the type of interface changed the vividness and location of the tingling in the foot. For both sockets, wearing the patch, the tingling in the foot was more vivid and distributed throughout the foot rather than being concentrated or more intense in the forefoot (Table 1). With the semi-rigid socket with a patch, the participant also perceived the arch of the foot, whereas he did not perceive it in the other three conditions. The localization and intensity of the RS were stable. Concerning the calf contractions, wearing the patch, the participant perceived his calf much more vividly, both through an increase in the felt contraction intensity and the distinction of the parts of the calf. Moreover, for both sockets, the patch induced sensations of calf contractions that varied with the gait cycle. Indeed, when the prosthetic heel hit the ground, he felt a contraction at the distal part of the phantom calf that moved upward toward the end of the stance phase. This gave him a sensation in the phantom calf that varied simultaneously with the progress of the stance phase. So, the sensations in the calf were dynamic, which was not the case for the calf without a patch and for the foot. When asked to rank the 4 conditions, the participant had a clear preference for the semi-rigid socket with patch and even asked to keep the patch at the end of the experiment.

**Table 1:** Description of the participant's phantom sensations according to the 4 prosthetic interface conditions.

	**Rigid**		**Semi-rigid**	
	**Without patch**	**With patch**	**Without patch**	**With patch**
Foot tingling	Restricted to the hallux Constant	Diffused over the toes and the heel Constant	Diffused over the foot, forefoot most intense Constant	Diffused over the whole foot Constant
Calf contractions	Global Constant	Focal Dynamic	Global Constant	Focal Dynamic
Ranking	4	3	2	1

*The detailed elements are the location and evolution during gait cycles of foot tingling and calf muscle contractions. “Constant”: Non-varying intensity and localization during gait cycles. “Dynamic”: Intensity and localization consistently varying during gait cycles. The last row contains the participant's ranking of the interface conditions, with 1 being the preferred*.

**Figure 8:**
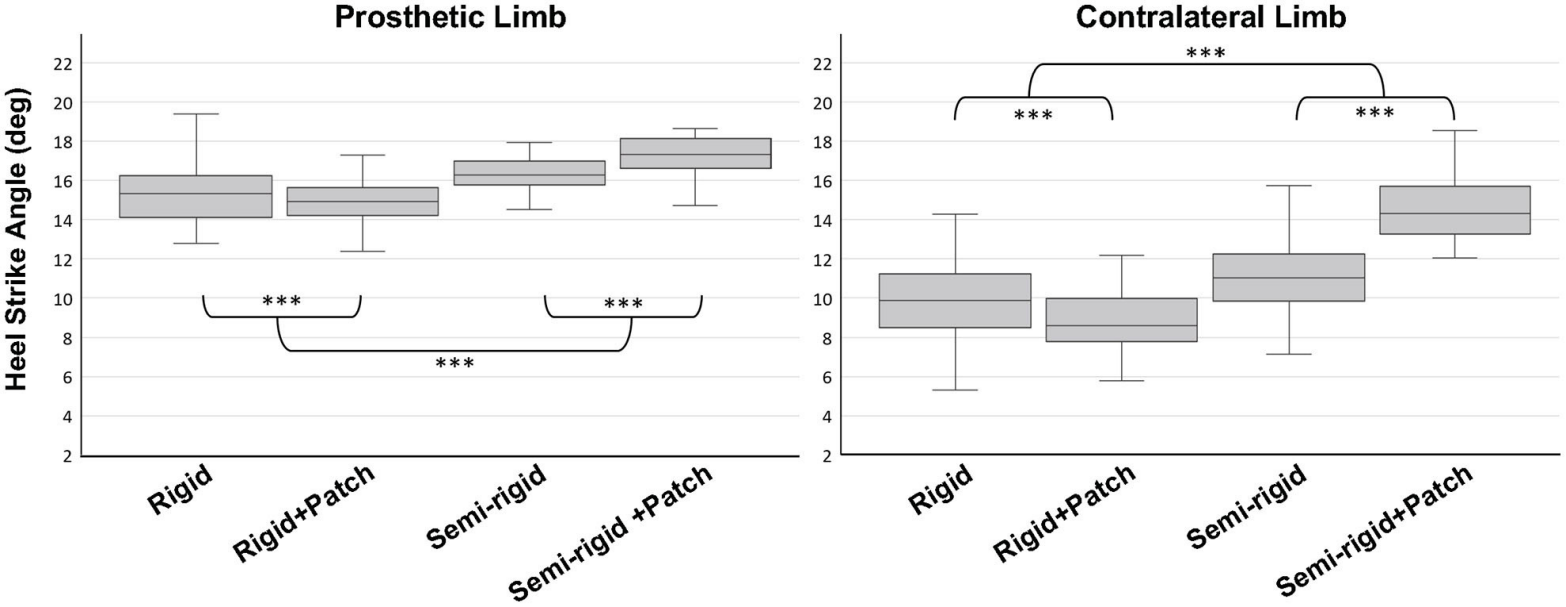
Heel strike angle in the 4 prosthetic interface conditions for the prosthetic (left) and the contralateral limb (right). For both limbs, the effect of the patch on the heel strike angle depended on the socket type (*p* < 0.001). ***p < 0.001.

Concerning the gait analysis, the type of socket and the presence of the patch influenced the **heel strike angle** similarly for both the prosthetic and the contralateral limb (Figure 8). Whether the patch was present or not, this angle was found to be larger for the semi-rigid socket than for the rigid one (*p* < 0.001). But the effect of the patch was different depending on the type of socket (*p* < 0.001). Indeed, the patch in the rigid socked decreased the heel strike angle, whereas in the semi-rigid socket it increased this angle (prosthetic limb: *p* < 0.05; contralateral limb: *p* < 0.001). Note that the condition preferred by the participant (semi-rigid socket with patch) had the largest heel strike angle for both limbs.

The interface condition influenced the **toe-off angle** differently for the prosthetic and the contralateral limb. For the prosthetic limb, regardless of the presence of the patch, the semi-rigid socket induced a larger angle than the rigid one (*p* < 0.05). For both types of sockets, the presence of the patch increased the angle (*p* < 0.001). So, the toe-off angle for the prosthetic limb was largest for the participant's preferred condition. For the contralateral limb, only the type of socket influenced the toe-off angle that was smaller when wearing the semi-rigid socket (*p* < 0.001).

Finally, both the type of socket and the presence of the patch influenced the **duration of the double support** phase. For both types of sockets, the presence of the patch diminished the duration (*p* < 0.05). Regardless of the presence of the patch, the semi-rigid socket induced a longer duration (*p* < 0.001).

## Discussion

### Key Results

This single case preliminary study showed that phantom sensations during walking can be modified by interventions on the socket interface. The participant reported that the nature of the phantom sensations and the concerned segments varied among the prosthetic interface conditions. These modifications were accompanied by changes in some spatiotemporal parameters.

### Limitations

Rehabilitation professionals and a few rare articles studying the link between the prosthetic interface and walking, acknowledge that mechanical stresses related to the socket influence walking (28) without even considering the phantom limb. Thus, although it seems likely, given the participant feedback, we cannot at this time assert that the changes in gait parameters are causally related to RS. The inclusion of a group of amputee participants without phantom sensations, for whom the same mechanical modifications on the interface of the prosthesis will be performed, will allow us to determine whether the phantom sensations modified by the interface did indeed influence the gait parameters or whether the modifications to the interface alone are responsible for this phenomenon.

Even if the modifications of the interface prove to be the cause of the modifications in the gait parameters, this would not affirm an increase in the quality of his gait. As the aim of the study was not to understand the walking strategy, we only recorded a limited number of spatiotemporal parameters. This does not allow us to assert that the entire gait strategy was modified by the intervention on the socket and phantom sensations, nor to explain the observed changes in certain spatiotemporal parameters, let alone their possible consequences in the long term. But even if we had recorded more parameters, the current state of knowledge on prosthetic locomotion does not allow us to define the optimal gait in lower limb amputees. This is why further analysis of gait but also of more cognitive variables such as mental workload (29), will allow us to determine whether (dynamic) phantom sensations can be used as somatosensory feedback and be useful for walking.

### Interpretation

The sensations induced by the patch were not simply the sum of the sensations caused by the individual pressures on areas 8 and 18 of the map. Indeed, the patch induced a more complete phantom foot, and the calf sensations became dynamic. This could be related to the fact that, first, the mapping was performed by delivering a focal pressure on the residual limb, whereas a socket induces a global pressure on the whole residual limb. Second, the mapping was performed at rest, whereas the effect of the interventions on the RS concerned walking. This induced highly dynamic variations in the pressure distribution in the socket, and thus cyclically altered the amount of additional pressure applied by the patch on the residual limb. The participant in this study already had many permanent phantom sensations during walking, which he reported using daily. However, we found an intervention that created *dynamic* phantom sensations in the calf varying systematically with the phases of the gait cycle. These dynamic sensations were particularly positive for the present participant who evaluated the conditions with the patch on top.

Evaluations of spontaneous phantom limb sensations (nature and localization) and the stimulation type and areas on the residual limb inducing RS are necessary to propose adapted modifications of the socket interface. For the participant of the present study, the stimulus inducing RS appeared to be pressure, but it is known that RS can be induced by other-than-static-pressure types of stimuli such as light touch (23), vibration (25), or electrical stimulation (20, 25). These stimuli have already been used to give feedback about phases of the gait cycles (11, 30). Yet, in these substitutive solutions, the stimulations were not located on the referred sensation map and thus gave only rise to a perception of the stimulus and not of the limb in motion. This means that the association between the nature of the stimulus and what it is supposed to represent must be learned. This is not the case in our approach that allows restoration of the perception of the limb evolving in action through RS. Our new approach could use similar gait phase detection and stimulation techniques as in substitutive solutions, but if the stimuli are delivered respecting the referred sensation map, they will induce the perception of the limb in action without needing a learning phase.

The modifications on the interface not only had a clear impact on the participant's phantom sensations but were also accompanied by a change in gait parameters. Indeed, the presence of the patch and the type of socket had a complex effect on several spatiotemporal gait parameters with (i) the influence of the patch depending on the type of socket, and (ii) the influence differing between the contralateral and prosthetic limb. Interestingly, the participant naturally expressed a strong preference for the semi-rigid socket with patch condition, which was the condition found with the most extreme values of the gait parameters among all conditions. Yet, the relationship between RS and the change in gait parameters is not clear. It is questionable whether the participant changed his gait parameters because the perception of his phantom leg in action allowed him to do so, or, on the contrary, because he liked the sensations during walking and the change in gait parameters allowed him to have them. The increase in heel strike and toe-off angles could be in favor of the second hypothesis, as this could have resulted in greater pressure on the proximal posterior part of the socket, which was the area where the patch was positioned.

Overall, this study suggests that after ower limb amputation, it is possible to restore the perception of the limb evolving in action through RS by modifying the design of the socket. Currently, the form of the socket is only considered in relation to support constraints but this new approach may lead to rethinking the personalization of the interface in terms of its relationship with the phantom limb. We encourage therefore to consider phantom sensations from the beginning of rehabilitation.

### Generalizability

As this preliminary study was based on a single case, our interpretations and conclusions cannot yet be generalized to the entire population of lower limb amputees. However, this feasibility study of the method of modifying phantom sensations perceived during walking is encouraging and now allows us to continue this research work with a larger population by improving the methodology taking into account the identified limitations.

## Data Availability Statement

The raw data supporting the conclusions of this article will be made available by the authors, without undue reservation.

## Ethics Statement

Ethical approval was not provided for this study on human participants because this was a pilot experiment to determine whether an in-depth study should be set up. This is done in total agreement with the participant and under supervision of the medical staff of the rehabilitation clinic following the patient. The orthoprosthetist of the patient did the interventions on the prosthesis. Given the results that we obtained, the ethical approval is in preparation in order to have a ethical review for the future study involving more patients. The participant provided his written informed consent to participate in this study.

## Author Contributions

LB performed all experiments and wrote the first version of the paper. SL conceived the prostheses sockets and together with CM helped during the experiments. SM helped analyzing the gait parameters. AT, IL, and JP contributed to theoretical considerations. JD is the senior researcher leading the project and finished the article together with LB. All authors contributed to the article and approved the submitted version.

## Funding

The funds received for open access publication come from the Institute of Movement Sciences, UMR7287, AMU-CNRS.

## Conflict of Interest

The authors declare that the research was conducted in the absence of any commercial or financial relationships that could be construed as a potential conflict of interest.

## Publisher's Note

All claims expressed in this article are solely those of the authors and do not necessarily represent those of their affiliated organizations, or those of the publisher, the editors and the reviewers. Any product that may be evaluated in this article, or claim that may be made by its manufacturer, is not guaranteed or endorsed by the publisher.
